# The association of depression with metabolic syndrome parameters and malondialdehyde (MDA) in obese women: A case-control study

**DOI:** 10.34172/hpp.2021.62

**Published:** 2021-12-19

**Authors:** Elnaz Vaghef-Mehrabani, Azimeh Izadi, Mehrangiz Ebrahimi-Mameghani

**Affiliations:** ^1^Postdoctoral Associate, Department of Pediatrics, Cumming School of Medicine, University of Calgary, Canada; ^2^Department of Biochemistry and Diet Therapy, School of Nutrition & Food Sciences, Tabriz University of Medical Sciences, Tabriz, Iran; ^3^Nutrition Research Center, Department of Biochemistry and Diet Therapy, School of Nutrition & Food Sciences, Tabriz University of Medical Sciences, Tabriz, Iran

**Keywords:** Insulin resistance, Major depressive disorder, Malondialdehyde, Metabolic syndrome, Obesity, Oxidative stress

## Abstract

**Background:** There is evidence for a bidirectional association between obesity and depression, and obesity is the main risk factor for metabolic syndrome (MetS). This study aimed to compare oxidative stress and MetS features between depressed and non-depressed obese women and study the association of depressive symptoms, oxidative stress, and components of MetS.

**Methods:** In this case-control study conducted in Tabriz (East Azarbaijan, Iran), obese women (body mass index [BMI]: 30-40 kg/m^2^ ) with a primary diagnosis of major depressive disorder (MDD; based on diagnostic interview with a psychiatrist; n=75) and their age-matched non-depressed controls (n=150) were enrolled. Beck Depression Inventory-version II (BDI-II) was used to assess depressive symptoms in both groups. Anthropometric parameters, blood pressure, fasting blood sugar (FBS), lipid profile and malondialdehyde (MDA) were measured.

**Results:** No significant differences in anthropometric parameters and blood pressure were observed between the two groups. However, FBS of the MDD group was significantly higher than the control (*P* <0.05). FBS was significantly correlated with BDI-II scores (r=0.158, *P* =0.017). No significant difference in lipid profile was observed between the groups. Serum MDA level was significantly lower in the MDD group and was inversely associated with BDI-II scores (r=-0.328, *P* <0.001). Overall, MDD was not significantly associated with MetS in our study (OR=0.848, 95% CI: 0.484, 1.487; *P* =0.566).

**Conclusion:** Although we found a correlation between higher depressive symptoms and some adverse metabolic outcomes, our findings do not support a significant association between MDD and MetS.

## Introduction

Depression and obesity are bidirectionally linked. This link can be explained by a number of factors. On one hand, people with obesity might be more susceptible to depression, probably due to reduced mobility, lower quality of life, and problems related to social bias and stigma.^1^ On the other hand, depression elevates the risk of metabolic syndrome (MetS) and obesity possibly through appetite changes, fatigue, and decreased activity.^2,3^ Besides, depression might make MetS more likely to occur due to poor health-related behaviors.^4^ There are several potential pathophysiological mechanisms to explain the relationship between obesity and depression including mutual inflammatory pathways.^5^ Another biological mechanism involved in the link between depression and MetS and its components is increased oxidative stress,^6^ a state of antioxidant and pro-oxidant imbalance, where the result is high levels of reactive free radicals.

Studies investigating the association between depression and MetS have come up with inconsistent results. In some studies, no relationship was found between these two disorders,^7,8^ while in the others, a direct link between them was reported.^9^ Furthermore, a relationship has been observed only between certain features of MetS and depression in some studies.^10^ Also, gender may modify the association between depression and MetS, even though studies have reported conflicting results regarding the moderating effect of sex on the connection between depression and MetS.^11-13^ For example, a study from Norway observed no effect of gender,^14^ while a study in Japan reported a link between MetS and depressive symptoms only in men.^11^ On the contrary, some studies have shown the association only in women.^12,13^

Depression is a mental health disorder influenced by multiple factors, and results from a single study carried out in a particular location may not apply to other people with different ethnicity, culture, sex, and age. Although depression prevalence is relatively high in Iranian adults,^15^ few studies have investigated its relationship with the components of MetS in this population. Thus, this study was designed to compare oxidative stress and MetS features between depressed and non-depressed obese women and assess the association of depressive symptoms, oxidative stress, and components of MetS in this population.

## Materials and Methods

### Study design and participants 

This case-control study was carried out from June 2018 to September 2018 in Tabriz, Iran. Seventy-five obese women (body mass index [BMI] between 30 and 40 kg/m^2^) aged 20-50 years with a primary diagnosis of major *depressive*disorder (MDD) and their age- and BMI-matched controls (n = 150) were enrolled. The sample size was calculated based on the results reported by Sotoude et al^16^, a confidence interval (CI) of 95%, and power of 80% using Power analysis and sample size software (PASS; NCSS, LLC, US) version 15. The sample size was estimated at 72 for the depressed group, and 144 for the control group. Taking into account a probable dropout, 75 and 150 depressed and non-depressed subjects were recruited in the case and control group, respectively. Diagnosis of MDD was confirmed through a diagnostic interview with a psychiatrist, and according to the DSM-5 (Diagnostic and statistical manual of mental disorders, 5th Edition) criteria.

All the patients were taking antidepressants in the last 6 months. The exclusion criteria of the study were as follows: thyroid dysfunctions, addiction to alcohol, cigarette smoking or drugs use, other major psychiatric or neurological diseases, pregnancy or lactation, and taking antioxidant supplements within the past two months. Those who had attempted weight loss during the past year were not included.

### Data collection

Data on demographic characteristics of the participants (age, marital status, occupation, education, and medical/medication history) was collected via face-to-face interviews. Body weight (to the nearest 0.1 kg) and height (to the nearest 0.5 cm) were measured using a Seca digital scale (Hamburg, Germany), with minimal clothing and no shoes on. BMI was calculated as weight (kg) divided by height squared (m^2^). Waist circumference (WC) was measured at midpoint between the distal border of the lowest rib and the superior border of the iliac crest. Hip circumference (HC) was measured at the greatest extension of the buttocks, as observed from the side. Both WC and HC were measured to the nearest 0.1 cm, using a non-stretch plastic tape.

Beck Depression Inventory-II (BDI-II) is a 21-item self-administered scale of depression and was completed by the participants. Each item consists of a response set of four sentences describing the extent of depressive symptoms during the preceding two weeks; scoring is based on Likert scale [0 for the first statement (absent or mild), and 3 for the fourth statement (severe)]. A total score is calculated by adding up the scores for all the items (range: 0-63); scores ≤13, and ≥29 indicate minimum, and severe depression, respectively; scores 14-28 specify mild-moderate depression. Validity and reliability of BDI-II has been confirmed among Iranian subjects [concurrent validity (Pearson correlation coefficient with Automatic Thoughts Questionnaire) = 0.77; internal consistency (Cronbach’s α) = 0.87; test-retest reliability (r) = 0.74].^17^

To control for the confounding effects of physical activity, the International Physical Activity Questionnaire-Short Form (IPAQ-SF) was completed by the patients. This questionnaire consists of seven questions inquiring about the type and duration of activities performed during a week prior to the interview. The participants were asked how often they spent a minimum of 10 minutes on vigorous activities (e.g., heavy lifting, digging, aerobic exercises, fast bicycling), moderate activities (e.g., carrying light loads, bicycling at regular pace, or doubles tennis), or walking (for any reasons at work and home, and for leisure) during the previous week. Total metabolic equivalents (MET-minutes/week) score was calculated for the subjects using an Excel program and according to the manual. An average METs of 3.3, 4.0, and 8.0 was considered for walking, moderate, and vigorous activities, respectively; then, these values were multiplied by the minutes spent on each of the activities and summed up to calculate the overall METs-minute score for the week. The participants were categorized as having high (MET-minutes/week of ≥3000), moderate (MET-minutes/week of 600-3000), or low (MET-minutes/week of < 600) level of activity.^18,19^

An aneroid sphygmomanometer (ALP K2, Japan) and stethoscope (ALP K2, Japan) were used to measured blood pressure. In a comfortably seated position with their legs uncrossed, and their feet on the floor and after 10 minutes of rest, two readings were taken five minutes apart, and the mean was recorded for each participant. After 12-hour overnight fasting, 10 ml of venous blood was drawn from the study participants and immediately centrifuged at 3500 rpm for 10 minutes. The supernatant serum was removed, aliquoted in microtubes, and stored at -80°C until assay. Serum fasting blood sugar (FBS), total cholesterol (TC), triglyceride (TG); and high-density lipoprotein (HDL) were measured through enzymatic methods using a colorimetric technique, by commercial kits (Pars-Azmoon Co., Tehran, Iran). LDL-C was calculated by Friedewald equation.^20^ The levels of malondialdehyde (MDA) were measured using spectrophotometry method.

### Statistical analysis

All statistical analyses were conducted using SPSS version 20.0 software (SPSS Inc, Chicago, IL, USA). The normality of data distribution was evaluated using Kolmogorov-Smirnov test. Independent samples *t* test and chi-square test were used to compare the results between the two groups. Pearson’s test was used to assess the correlation between BDI-II scores and MetS components as well as MDA. Logistic regression was used to calculate OR 95% CI for the association between MDD and MetS. Since the outcome (dependent) variable needs to be dichotomous in Logistic Regression, we first categorized study subjects into two groups: with MetS and without MetS. Presence or absence of MetS was determined based on the measured MetS components and according to the National Cholesterol Education Program (NCEP) Adult Treatment Panel III (ATP III) definition, for each participant. We used a different Iranians-specific WC cutoff point than the NCEP/ATPIII criteria to define high WC. A woman must meet at least three criteria of the following five to be categorized as having MetS: WC > 90 cm; TG > 150 mg/dL; HDL-C < 50 mg/dL; SBP > 130 mm Hg or DBP > 85 mm Hg; FBS > 100 mg/dL.^21,22^ A two-sided *P* < 0.05 was considered statistically significant.

## Results

The general characteristics of the participants are presented in Table 1. The means (SD) of the age for the MDD and control groups were 39.64 (SD 7.70) and 39.97 (SD 7.58) years, respectively. In both groups, the majority of the participants were married. The women with MDD had significantly higher education levels than non-depressed (*P* < 0.001). In addition, the majority of the individuals with MDD were not occupied.

**Table 1:** Baseline characteristics of the study participants

**Variables**	**MDD** **(n=75)**	**Non-MDD** **(n=150)**	***P*** ** value**
Age (years)	39.64 (7.70)^a^	39.97 (7.58)	0.762^c^
BDI-II score	21.99 (8.55)^a^	7.56 (3.31)	< 0.001^c^
Marital status			0.062^d^
Single	3 (4.00)^b^	14 (9.30)	
Married	70 (93.30)	136 (90.70)	
Divorced or widow	2 (2.60)	0 (0.00)	
Education			
Illiterate	2 (2.70)	3 (2.00)	< 0.001^d^
Diploma and lower	52 (69.30)	142 (94.70)	
Bachelors and higher	21 (28.00)	5 (3.30)	
Occupation			0.017^d^
Housewife	64 (85.30)	107 (71.30)	
Employee	9 (12.00)	22 (14.70)	
Self-employed	2 (2.70)	21 (14.00)	
Physical activity			0.226^d^
Low	71 (94.70)	147 (98.00)	
Moderate	4 (5.30)	3 (2.00)	

Abbreviations: BDI, Beck depression inventory; MDD, major depressive disorder. ^a^ Mean (SD); ^b^N(%); ^c^ Independent-samples ^t^ test; ^d^ Chi-square test.

Overall, 8 (10.7%) patients in the MDD group received citalopram, 30 (40.0%) patients used sertraline, and 37 (49.3%) patients took fluoxetine as the main pharmaceutical treatment for their depression. The median duration of antidepressant therapy was 4.00 (2.75, 6.00) years.

As depicted in Table 2, no significant differences in anthropometric parameters and blood pressure were observed between the groups. However, FBS of the MDD group was significantly higher than the control (*P*< 0.05). Additionally, FBS showed a weak but significant negative association with BDI-II depressive score (r = 0.158, *P* = 0.017; Table 3). No significant difference in lipid profile was observed between the groups (Table 2). Serum MDA level was significantly higher in the control group (Table 2), and was inversely associated with BDI-II depression score (r = -0.328, *P* < 0.001; Table 3).

Forty-two and 90 subjects had MetS based on the NCEP/ATPIII criteria, in the MDD and control group, respectively. No significant association was found between MDD and MetS (OR = 0.848, 95% CI 0.484, 1.487; *P* = 0.566).

**Table 2:** Comparison of MetS components and oxidative stress between obese women with and without Depression

**Variables**	**Depressed** **(n=75)**	**Non-depressed** **(n=150)**	***P*** **value** ^a^
Weight (kg)	87.49 (11.92)*	87.28 (9.94)	0.888
BMI (kg/m^2^)	34.18 (3.79)	34.51 (2.94)	0.516
WC (cm)	109.52 (11.18)	109.55 (9.51)	0.983
HC (cm)	118.19 (12.52)	116.65 (7.93)	0.262
WHR	0.93 (0.07)	0.94 (0.07)	0.305
SBP (mm Hg)	117.20 (13.95)	115.20 (13.82)	0.309
DBP (mm Hg)	78.47 (10.59)	77.15 (9.43)	0.344
FBS (mg/dL)	86.49 (14.60)	82.67 (11.01)	0.029
TG (mg/dL)	162.99 (82.03)	155.49 (59.68)	0.483
TC (mg/dL)	205.89 (37.63)	204.79 (33.44)	0.824
HDL (mg/dL)	49.52 (10.19)	48.77 (9.79)	0.595
LDL (mg/dL)	123.78 (36.04)	124.92 (31.11)	0.805
MDA (nmol/mL)	1.46 (0.41)	1.91 (0.42)	< 0.001

Abbreviations: BMI, Body mass index; DBP, Diastolic blood pressure; FBS, Fasting blood sugar; HC, Hip circumference; HDL, High-density lipoprotein; LDL, Low-density lipoprotein; MDA, Malondialdehyde; MetS, Metabolic syndrome; SBP, Systolic blood pressure; TC, Total cholesterol; TG, Triglyceride; WC, Waist circumference; WHR, Waist to hip ratio. * Mean (SD); ^a^ Independent-samples *t*test.

**Table 3:** Correlation of BDI-II scores with Metabolic Syndrome features and serum MDA (n = 225)

**Variable**	**r** ^a^	***P*** ** value**
WC (cm)	0.076	0.255
SBP (mm Hg)	-0.036	0.596
DBP (mm Hg)	-0.003	0.965
FBS (mg/dL)	0.158	0.017
TG (mg/dL)	0.007	0.912
HDL (mg/dL)	-0.017	0.802
MDA (nmol/mL)	-0.328	< 0.001

Abbreviations: BDI-II, Beck Depression Inventory-II; DBP, diastolic blood pressure; HDL, High-density lipoprotein; LDL, Low-density lipoprotein; MDA, Malondialdehyde; SBP, Systolic blood pressure; TC, Total cholesterol; TG, Triglyceride; WC, Waist circumference. ^a^ Pearson's correlation coefficient.

## Discussion

In the present study, the relationship between depression, the components of MetS and oxidative stress were investigated, and these parameters were compared between obese women with and without MDD.

We found that serum FBS levels were significantly higher in depressed vs. non-depressed individuals, and that serum FBS levels were positively correlated with depression scores, suggesting an interaction between depression and metabolic dysregulation. Depression is reported to adversely impact lifestyle behaviors including diet, exercise, and smoking^4^; which might be associated with metabolic abnormalities including increased blood sugar. It is noteworthy that people experiencing depressive symptoms may not adopt healthy lifestyles, which can subsequently result in metabolic disorders, which in turn might aggravate depression symptoms.^23^ In addition, shared underlying mechanisms between depression and metabolic dysregulations, and the joint effects of these two disorders might augment the risk of diabetes development.

There is evidence of dysfunction of the hypothalamic-pituitary-adrenocortical axis and sympathetic nervous system activation in depression, which can lead to abdominal fat accumulation and impaired glucose metabolism.^24^ In addition, weight gain associated with some antidepressant drugs may increase the risk of diabetes.^25^ Increased inflammation is also implicated in both depression and metabolic dysregulation.^26^ Thus, each one of these two disorders can trigger the other, which in turn contributes to a vicious cycle. Adherence to a healthy lifestyle is required to break this cycle.

In this study, no difference was observed between depressed and non-depressed controls in terms of other features of MetS including blood pressure, lipid profile, or WC. This could be due to the fact that both groups showed dyslipidemia (characterized by TG > 150 mg/dL, TC > 200 mg/dL, and HDL < 50 mg/dL). Indeed, the proportion of the subjects with high WC ( > 88 cm) was markedly high in both groups. These findings are consistent with other studies that showed no correlations between the components of Mets and depression.^16,27^ In the study by Sotoudeh et al,^16^ WC was significantly higher in those recently diagnosed with depression compared to the other subjects. However, Amini et al,^28^ reported that older adults with depressive symptoms had lower BMI, WC, serum LDL-C and TG than healthy controls. Similar to our findings, a recent study,^29^ reported no correlation between depressive symptoms and blood pressure in women, while a negative association was found for men. It seems that depression duration, undergoing treatment, use of different tools to define or measure depression, age differences in study samples, confounding factors including diet, smoking, and physical activity account for the inconsistent results. Moreover, the effects of gender in the pathophysiology of depression and hormonal status may account for the null findings among women.^30,31^ More studies are needed to address the potential impacts of gender in the association of depression and MetS components.^31^

The current study provides support for decreased levels of MDA in obese women with depression. A recent systematic review reported an association between higher levels of biomarkers of oxidative stress and depressive disorder.^32^ Previous studies that measured MDA levels in depression included different types of depression such as recurrent depression, the drug naive first episodes, and comorbid disorders; most of them have represented high MDA levels in depression. Indeed, MDA has been suggested as a novel predictive biomarker for depression.^33,34^ Camkurt et al,^33^ reported increased concentrations of MDA in drug-free first-episodes patients with major depression. Likewise, another study found increased levels of MDA in depressed patients, compared with controls, and even a greater MDA concentration in patients experiencing a depression relapse.^35^ The elevated levels of urinary MDA has been associated with 30% increase in depressive symptom scores.^36^Interestingly, antidepressant treatment has been shown to significantly reduce serum MDA concentration,^37,38^ which might partially explain the lower levels of MDA in patients with MDD in our study, as all our patients with MDD were receiving standard antidepressant treatment. However, in a study by Rybka et al,^39^ MDA was also found at significantly elevated levels in depressed patients, regardless of whether or not they were receiving treatment. The prolonged oxidative stress and elevated MDA levels are implicated in major depression; high susceptibility of the brain to oxidative stress accounts for this association.^40^ Thus, the disturbed balance between antioxidant and pro-oxidant indicators needs to be restored. The MDA-lowering effects of antidepressant treatment seems to be duration-dependent; a longer duration is required to decrease MDA levels.^41^

To our knowledge, this is among few studies exploring the link between depression and MetS features, in which the case (MDD) group were receiving their standard treatment. Some limitations of our study should be noted. Considering the cross-sectional design and the relatively small sample size of this study, the casual relationship between depression and MetS could not be determined. Only patients with mild to moderate MDD were recruited in this study, and therefore, the findings may be less generalizable to the other category of MDD (severe). Furthermore, the influence of dietary intake on the association between depression and MetS was not considered in this study.

## Conclusion

Although we showed an association between depressive scores and some MetS features, findings from this study did not support a significant association between MDD and MetS. Depression and MetS might have a reciprocal and bidirectional association. However, antidepressants may be associated with reduced oxidative stress in patients with depression. Therefore, the pharmacological effects of the medications for MDD treatment on metabolic factors should be taken into account when investigating the association of MDD and MetS. In addition, future studies are warranted to investigate the mechanisms underlying the potential association between depression and obesity, cardiometabolic risk and inflammation, and explore the effects of targeted interventions on depressive symptoms and cardio-metabolic risk.

## Acknowledgments

We sincerely thank those who participated in this study.

## Funding

This work was funded by the “Research Vice-Chancellor” and “Nutrition Research Center” of Tabriz University of Medical Sciences, Tabriz, Iran.

## Competing interests

The authors declare that they have no competing interests.

## Ethical approval

The present study was conducted in accordance with the Helsinki Declaration of 1975, as revised in 2008. The study participants were given a detailed explanation of the study procedures, and they signed an informed consent form. The study protocol was approved by the ethics committee of Research Vice-Chancellor of Tabriz University of Medical Sciences (Tabriz, Iran; Ethics code: IR.TBZMED.REC.1396.1077).

## Authors’ contributions

EVM and MEM conceptualized the study. EVM collected the data. EVM and AI analyzed the data and MEM contributed to interpretation of the results. EVM and AI prepared the first draft of the paper and subsequent reviews and revisions were completed by all authors. All authors reviewed the final draft and gave approval to publish. All authors agreed to be responsible for all aspects of the work in ensuring that questions related to the accuracy or integrity of any part of the work are appropriately investigated and resolved.
